# Shade is the most important factor limiting growth of a woody range expander

**DOI:** 10.1371/journal.pone.0242003

**Published:** 2020-12-02

**Authors:** David Ward

**Affiliations:** Department of Biological Sciences, Kent State University, Kent, OH, United States of America; Technical University in Zvolen, SLOVAKIA

## Abstract

The expansion of woody plants into grasslands and old fields is often ascribed to fire suppression and heavy grazing, especially by domestic livestock. However, it is also recognized that nutrient availability and interspecific competition with grasses and other woody plants play a role in certain habitats. I examined potential factors causing range- and niche expansion by the eastern redcedar *Juniperus virginiana*, the most widespread conifer in the eastern United States, in multifactorial experiments in a greenhouse. Historical records suggest that the eastern redcedar is a pioneer forest species, and may be replaced as the forest increases in tree density due to shading. Another possible factor that affects its distribution may be nutrient availability, which is higher in old fields and other disturbed lands than in undisturbed habitats. In its historic range, eastern redcedars are particularly abundant on limestone outcrops, often termed ‘cedar barrens’. However, the higher abundance on limestone could be due to reduced interspecific competition rather than a preference for high pH substrates. I manipulated shade, fertilization, lime, and interspecific competition with a common dominant tree, the post oak *Quercus stellata*. In a separate experiment, I manipulated fire and grass competition. I measured growth rates (height and diameter) and above- and belowground biomass at the end of both experiments. I also measured total non-structural carbohydrates and nitrogen in these plants. Shade was the most important factor limiting the growth rates and biomass of eastern redcedars. I also found that there were significant declines in nitrogen and non-structural carbohydrates when shaded. These results are consistent with the notion that the eastern redcedar is a pioneer forest species, and that shade is the reason that these redcedars are replaced by other tree species. In the second experiment, I found that a single fire had a negative effect on young trees. There was no significant effect of competition with grass, perhaps because the competitive effect was shading by grasses and not nutrient depletion. Overall, the effects of shade were far more apparent than the effects of fire.

## Introduction

Native species are capable of spreading rapidly into new habitats and niches, and act similarly to invasive species, usually in response to a disturbance of that habitat [1–3]. However, invasive species were far more likely than native species to respond to disturbances [2]. Furthermore, the impacts of range expansion by native species are usually of a much lower magnitude than those of invasive plant species [2]. However, some authors consider there to be little difference between invasive species and native invasive (range-expanding) species in terms of their effects on the environment [4–6]. There clearly are negative effects of range-expanding native species on features of the environment, including alterations and reductions in diversity [6–13], diminished light reaching the understory [14], alterations in stream discharge and runoff patterns [15–19], altered soil nitrogen and carbon dynamics [20, 21] and reductions in arbuscular mycorrhizal fungal diversity [22].

A particularly problematic range-expanding species is the eastern redcedar *Juniperus virginiana* [23]. This species is the most widespread conifer in eastern North America, and is rapidly expanding both its niche [19, 20, 21, 24–29] and its range east of the 100^th^ parallel [30–34]. Encroachment of the eastern redcedar on the Great Plains of the United States may have exceeded the threshold of control back to natural prairie grassland [35]. There has been about an 80% increase in the abundance of the eastern redcedar across its range [6]. Possible causes of change in its niche, and ultimately in its range, include sustained heavy grazing by domestic livestock [36] and fire suppression [37], although the last-mentioned population increase may only occur during droughts (i.e., when insufficient water is available) [38]. This species has also expanded its niche through human plantings, for example, as a windbreak around houses or a shelterbelt around crop fields, as landscaping, and as wildlife habitat [22, 39, 40]. Eastern redcedar is also naturally expanding its niche *within* its historic range [9, 10, 41, 42]. Eastern redcedar abundance is increasing in terms of area occupied, number of locations, and tree size [12, 32–34, 43].

In addition to expanding into rangelands, where eastern redcedars reduce the amount of grazing land for cattle and sheep [9, 10, 44] and browsing for wild ungulates [3], this species causes serious alterations in ecosystem services. For example, reductions in stream run-off and discharge are widespread where eastern redcedar is abundant [15–18, 45, 46]. There is also evidence of alterations in nitrogen and carbon accumulation in eastern redcedar-encroached grasslands [21, 47], and little or no increase in carbon storage in eastern redcedar soils on the Great Plains [48]. Additional ecosystem services that are reduced by niche- and range-expansion of eastern redcedar are bird populations that are grassland-dependent [49, 50]. In general, there is little evidence of resistance to encroachment by eastern redcedar due to diversity of competing plants [51]. There are, however, considerable negative effects of eastern redcedar on the diversity of native plants [11, 14, 44, 52], resulting in near monocultures of eastern redcedar (termed the ‘green glacier’ [53]). The costs of removal of encroaching eastern redcedar are extremely high ([37, 53–58], so alternative means of reducing their densities need to be sought.

Despite the considerable concern over the range- and niche-expansion of eastern redcedars, the mechanisms behind its increases in abundance are poorly understood. While there have been many studies examining the consequences of heavy grazing and fire suppression on its range expansion, none have simultaneously examined the effects of factors such as shade, nutrient availability, competition, fire, and tree and grass competition. Furthermore, in many areas of the U.S. MidWest, eastern redcedar is particularly abundant in high-pH limestone glades, often termed ‘cedar barrens’ or ‘cedar glades’ ([57, 59–66]. However, the greater abundance of the eastern redcedar on limestone-rich soils than on other soils may be due to reduced competition there, rather than a preference for high pH *per se* [62, 67]. Also, eastern redcedars growing on limestone can be quite short as adults (2–4 m) while on deep, moist, well-drained alluvial sites they can often reach 16.5–18 m at 50 years of age, indicating that they grow better on high-nutrient soils [62, 68–70].

Partitioning of nitrogen and total non-structural carbohydrates (TNC) above- and belowground may determine whether there was resource re-allocation that was consistent with optimal partitioning theory (OPT) [71–76]. OPT predicts that plants should allocate biomass to those parts or organs that most limit growth [71, 75, 77]. Thus, plants living in shaded habitats should invest more in leaf surface area than plants living in open habitats [78] and plants living in low-nutrient soils should invest heavily in roots [75]. However, the generality of OPT has been questioned by several authors [76, 79–82]. For example, under high- and low-light conditions, variation in life-history traits (e.g., deciduous vs. evergreen) explained more of the variation in factors such as specific leaf area, specific root length and relative growth rate than could be explained by OPT [82]. Furthermore, much of the variation claimed to be explained by OPT may be driven by ontogenetic differences in plant size [73, 83–85]. I was particularly interested in ascertaining the effects of resource-allocation changes appropriate to the effects of shade, nutrient fertilization, fire and competition, such as increased investment in aboveground biomass in the shade and re-allocation of storage (particularly non-structural carbohydrates) to the roots in the case of low-nutrient soils and greater allocation of nitrogen to the aboveground parts of the plant when fertilized with nitrogen [72, 86–95].

I established two experiments to test the effects of the main factors affecting the establishment and growth of eastern redcedars:

Experiment 1: I manipulated the presence of shade, fertilizer, lime, and interspecific competition with a common co-dominant tree, the post oak *Quercus stellata*. I predicted that shade would have a significant negative effect on their growth, fertilization and lime addition would cause an increase in growth, and interspecific competition would lead to a decline in growth. Plants growing in shade should invest more in aboveground biomass, nitrogen and non-structural carbohydrates than unshaded plants [78, 92, 96]. Plants growing in low-nutrient soils should invest in belowground biomass and increased storage of non-structural carbohydrates [75]. Plants enduring interspecific competition should invest more in non-structural carbohydrates to buffer against negative effects of competition than plants not suffering from competition [95]. If eastern redcedars prefer to grow in high pH conditions (i.e. with lime), they should invest more in nitrogen and non-structural carbohydrates [97–99].

Experiment 2: I manipulated the presence of fire and grass competition. I predicted that fire would cause a decline in growth of eastern redcedars as would the presence of the grass competitor. Burned plants should invest in increased nitrogen and in belowground biomass [77]. Similarly, in eastern redcedars suffering from competition with grasses there should be increased development of belowground biomass and in nonstructural carbohydrate storage [75].

## Methods

### Experimental design and treatments

All eastern redcedar saplings were of similar size when purchased from Pineland's Nursery in Columbus, New Jersey and were about 18 months old. Mean initial heights of eastern redcedars ± S.E. at the start of the experiment on 23 June 2016 were 138.4 ± 2.54 mm, and mean initial stem diameters ± S.E. were 2.6 ± 0.06 mm.

In the first experiment, run from May 2016 until August 2018, I manipulated the levels of shade, fertilizer, lime, and competition with the post oak *Quercus stellata* in a greenhouse. I placed one eastern redcedar in each container (for further details, see below). I used Green-Tek^®^ knitted 80% shade cloth (BFG Supply, Burton, Ohio) and a control (unshaded). I checked the shading effect using an AccuPAR model LP-80 ceptometer (Decagon Devices, Inc., Pullman, Washington) in photosynthetically active radiation (PAR) in the 400–700 nanometer waveband during biweekly measurements over the study period (32 measurements during each period). I confirmed that there was a highly significant reduction in the effect of sunlight caused by the shade cloth (78.7% ± 0.53 reduction; minimum = 73% reduction; maximum = 84% reduction).

I fertilized half the containers with nitrogen at 30 g m^-2^ twice per year [100], and a control with half the fertilizer (15 g m^-2^ twice per year). The lower level of fertilization was used to minimize potential volatilization in case there was insufficient supply from the potting soil (Scott’s^®^ Hyponex Potting Soil). I also raised the level of alkalinity in the soil by adding lime to half the containers to simulate the commonly seen association of eastern redcedars with limestone habitats and a control. At the end of the experiment, there was a significant difference in soil pH of the lime treatment (mean pH = 5.7 ± 0.26) and controls (mean pH = 5.4 ± 0.35) (t = 5.498, p < 0.001). I also introduced competition with a common tree, the post oak *Quercus stellata*, in half the containers (one post oak per container), with the remainder being controls (no post oaks). My rationale was that it may not be lime (i.e., high pH *per se*) that causes eastern redcedars to be so abundant on cedar barrens but rather because there are relatively few competitors, particularly trees [59–61]. I used the post oak to test for interspecific competition because it is a dominant species over large areas of the midwestern U.S. [29, 65, 66], particularly in habitats where eastern redcedars are common. I purchased post oaks from Mossy Oak Nativ Nursery in Westpoint, Mississippi. Mean initial heights of post oaks were 195.6 ± 7.75 mm, and mean initial stem diameters were 4.6 ± 0.11 mm.

I used 95 L containers (n = 120) (depth = 70 cm; 55 cm diameter) so that the trees would not be constrained by soil availability. I used a split-plot experimental design, with shade (and control) the *whole* plot (replicated six times) and the *sub-plots* completely randomized and consisting of the remaining factors (nutrients, lime, and competition). Water availability was not manipulated and was provided *ad libitum* by means of drip irrigation.

In the second experiment, I tested for the effects of fire and grass competition on similarly-sized young trees (about 18 months old) for one month in a greenhouse. I applied the burning technique [101, 102] in which a blow torch was held about 20 cm from the tree and burned the entire height of each tree on two opposing sides (n = 30 burned trees). The other 30 trees were controls (unburned). I manipulated the presence of grass (*Bromus inermis*) using half the burned and half the control trees (n = 15 burned and 15 control). *Bromus inermis* is a weakly to strongly rhizomatous invasive C_3_ species that is now found in all states of the United States and in Canada [103–105]. Grasses were grown for 30 days before the onset of this experiment. The soil used was Promix^®^ Premier Ultimate Potting Mix. In this second experiment, I measured above- and belowground biomass. For the assessments of storage, I measured total non-structural carbohydrates using a standard protocol [93, 106, 107]. All of the analyses were done in a single laboratory because inter-laboratory comparisons have proved problematic in terms of repeatability [108]. I also recorded % nitrogen in the trees using a Rapid N Exceed^®^ Elementar nitrogen analyzer. I recorded tree condition as 1 (poor ~mostly dead) to 10 (good). There was a significant positive correlation between tree condition and total biomass (r = 0.55, p < 0.001).

### Statistical analysis

For the first experiment, I performed a general linear model for repeated measures for a split-plot design. The design was unbalanced and, consequently, a Type III model was employed. I first performed a MANOVA because of the large number of dependent variables. The dependent variables were mean relative growth rate (RGR) after one year (height and stem diameter; starting with initial height and stem diameter), and final mass (measured as total, aboveground and belowground), belowground total non-structural carbohydrates (TNC) and total nitrogen. The independent variables were shade (whole plot), nutrients, lime, and competition (all sub-plots). All variables were log_10_ transformed to fulfill the requirements of normality and homogeneity of variance.

For the second experiment, I used a MANOVA for a completely randomized design on RGR, final mass (aboveground and belowground), tree condition, non-structural carbohydrates (above- and belowground; also analyzed as the constituents of starch and soluble sugars [94, 95, 108], and total nitrogen (above- and belowground), with fire and grasses as the independent variables. I used final total mass (aboveground + belowground) as a covariate.

For the significant variables only, I used general linear models to test for main effects and interactions. These fire- and grass-experiment data, including tree-condition data, were normally distributed and were not transformed. I used SPSS v. 26 for all analyses.

## Results

### Shade, fertilizer, lime and oak competition experiment

**Fig 1:**
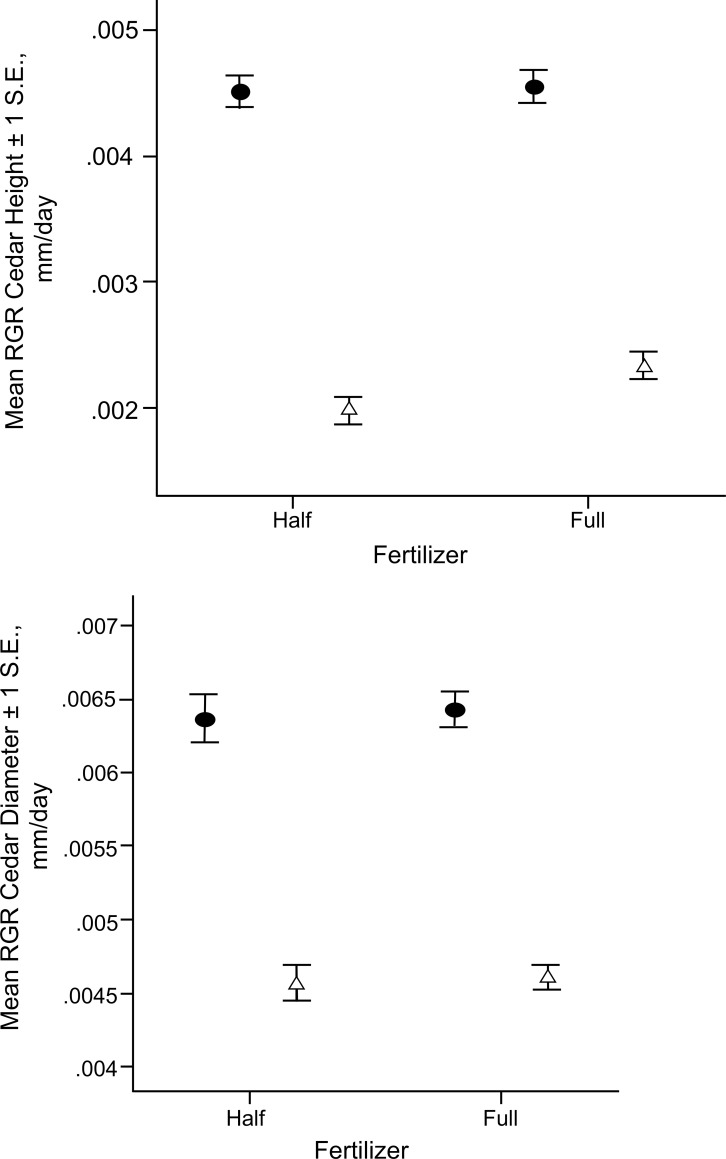
Mean relative growth rate (RGR) ± 1 S.E. of redcedars under half and full fertilization (30 g m^-2^) in unshaded (control) (filled circles) and 80% shade (open triangles). a) Mean RGR for height. b) Mean RGR for stem diameter. There was no significant main effect of fertilizer and no significant interaction between shade and fertilizer.

All plants survived this experiment. There was a significant effect of shade (whole-plot effect) on mean relative growth rate (RGR) (final-initial) for redcedar height (F = 464.158, p < 0.001) and stem diameter (F = 216.786, p < 0.001) (Fig 1A and 1B). There was no significant effect of the other main factors (fertilizer, lime, competition) nor interaction effects between these variables for RGR height and stem diameter (p > 0.05).

I found no significant effect of shade with regard to RGR for post-oak height (F = 1.619, p = 0.215) but there was a significant effect with regard to post-oak stem diameter (F = 22.373, p < 0.001), with the shade trees (6.4 ± 0.20 mm) having a smaller stem diameter than the controls (8.1 ± 0.47 mm). There were no significant main or interaction effects of fertilizer or lime on RGR for oak height and stem diameter (p > 0.05).

**Fig 2:**
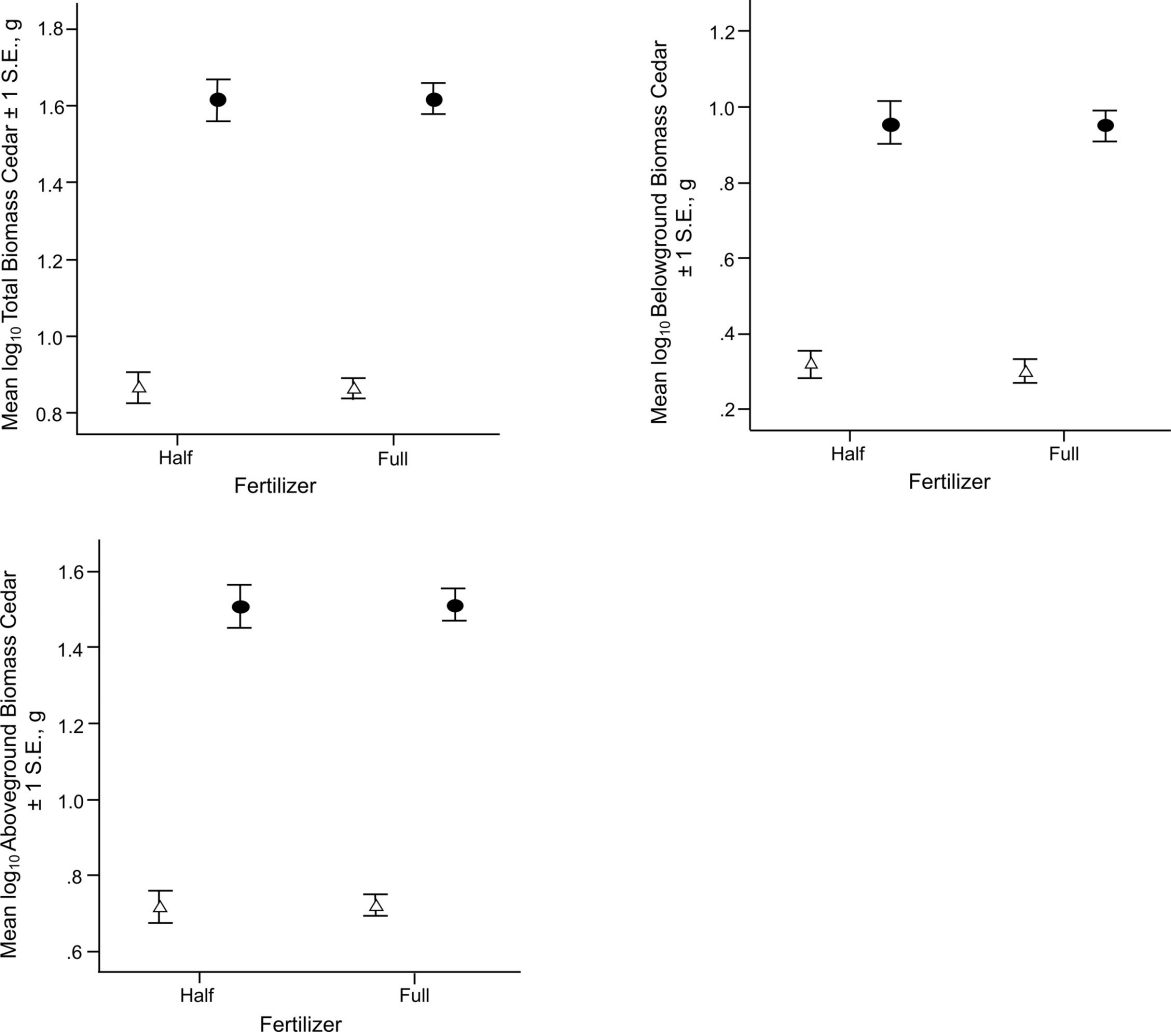
The log_10_ mean biomass ± S.E. of redcedars of half and full fertilization (30 g m^-2^) under control (closed circles) and shade (open triangles). a) Total biomass. b) Aboveground biomass. b) Belowground biomass. There were no significant interaction effects between shade and fertilizer nor a significant main effect for fertilizer.

There was a significant main effect of shade on log_10_ transformed total biomass (F = 353.975, p < 0.001), aboveground biomass (F = 357.079, p < 0.001) and belowground biomass (F = 247.147, p < 0.001) of eastern redcedars, with considerably higher values recorded for all unshaded plants (Fig 2A–2C). There were no other significant main or interaction effects, with the exception of a three-way interaction of shade X lime X competition on belowground biomass (F = 4.418, p = 0.041).

**Fig 3:**
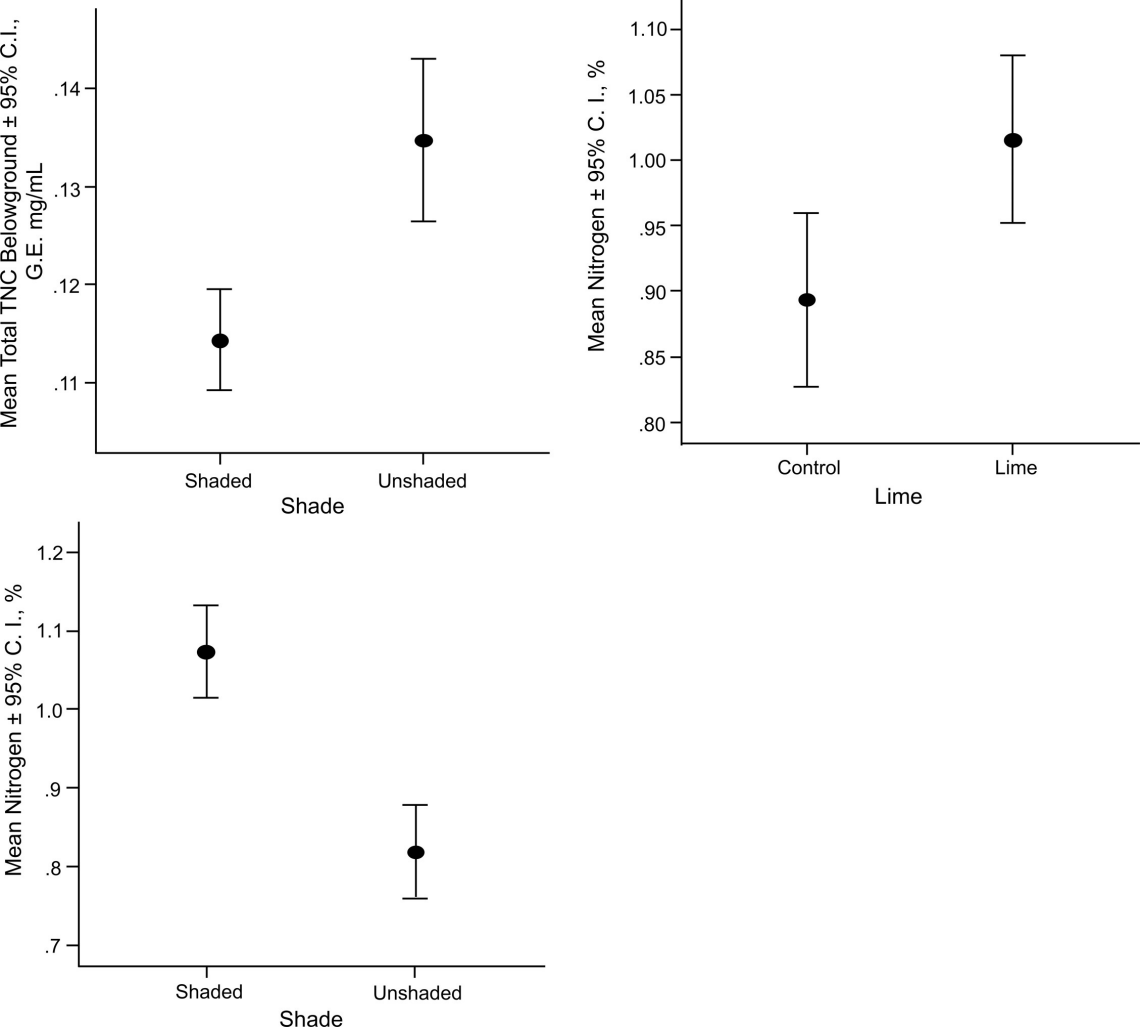
There were significant differences in (a) total non-structural carbohydrates (TNC) belowground and (b) total % nitrogen for shaded plants, but in opposite directions. Unshaded plants had higher concentrations of TNC and lower concentrations of nitrogen than shaded plants. There was also a significant main effect of lime addition on (c) nitrogen, with higher concentrations in plants receiving lime addition. G.E. = glucose equivalents. C.I. = 95% confidence interval.

There was a significant effect of shade on N (F = 20.082, p < 0.001) and a significant interaction effect of shade X lime X competition (F = 11.349, p = 0.001) but no other significant interactions or main effects. There was a significant effect of shade (F = 39.842, p < 0.001) and lime (F = 9.454, p = 0.003) on the concentration of nitrogen belowground (Fig 3). There were no other significant main or interaction effects.

### Burning and grass competition experiment

I recorded multiple dependent variables (tree height, trunk diameter, total biomass, aboveground biomass, belowground biomass, tree condition, nitrogen, and total non-structural carbohydrates (TNC)). Consequently, I used a MANOVA to control for Type I statistical error. There was a significant effect of burning in the MANOVA (Wilks’ λ = 0.580, p = 0.008). However, there were no significant effects of grass competition (Wilks’ λ = 0.706, p = 0.138), nor significant interaction effects between burning and grass competition (Wilks’ λ = 0.678, p = 0.082).

**Fig 4:**
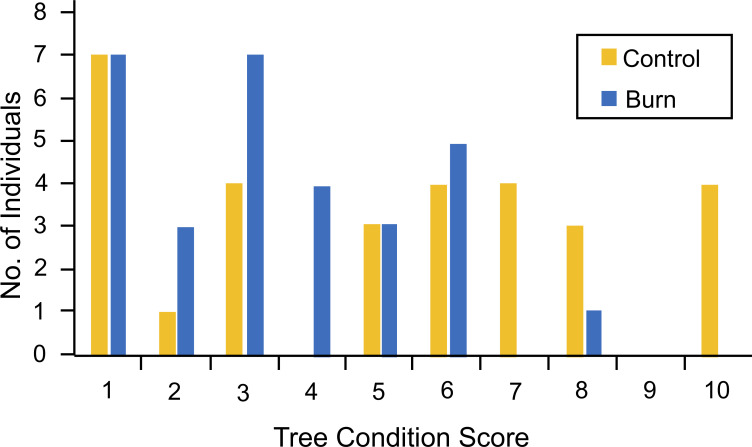
Comparison of tree condition between burned and control trees. 1 = poor condition, 10 = best condition. Median value for control trees = 5.5; median value for burned trees = 3. Note that as many control (unburned) trees had as low a tree-condition score as burned trees.

The univariate ANOVA showed that were neither significant differences in tree height (F = 1.038, p = 0.313) nor trunk diameter (F = 1.465, p = 0.231) between burned and unburned treatments one month after initiation of the experiment. There were significant effects for total biomass (F = 5.239, p = 0.026) and aboveground biomass (F = 6.388, p = 0.014), with the burned trees weighing less than the control (unburned) trees. However, there was no significant difference in belowground biomass between control and burned trees (F = 2.242, p = 0.140). There was a significant main effect for burning on tree condition (F = 5.328, p = 0.025) (Fig 4). All of the burned eastern redcedar trees were in relatively good condition one month after burning (mean condition ± S.E. = 3.43 ± 0.358) but their condition was not as good as the unburned control trees (mean condition ± S.E. = 5.07 ± 0.571).

There was also a significant reduction in total nitrogen (above- plus below-ground) in burned compared to unburned plants (F = 6.164, p = 0.016). There was a significant reduction in % N belowground in burned trees compared to unburned trees (F = 5.500, p = 0.023) (control trees = 1.17 ± 0.078%; burned trees = 0.88 ± 0.062%) but not % aboveground nitrogen (F = 2.419, p = 0.126).

There were no significant differences in soluble starches between burned and unburned plants both above- and belowground (aboveground: p = 0.503; belowground: p = 0.689), nor effects of grass competition (p > 0.05) nor interaction between burning and grass competition (p > 0.05). Similarly, there were no significant differences in soluble sugars or total non-structural carbohydrates (TNC) above- and belowground (p > 0.05).

Although there were no significant differences in TNC between burned and control trees (above-, belowground, and total biomass), larger trees stored more TNC than smaller trees, regardless of treatment. There was a positive correlation between TNC and log_10_ total biomass (burned trees: r = 0.59, F = 14.949, p < 0.001; control trees: r = 0.39, F = 5.091, p = 0. 033). Similarly, there was a positive correlation between % nitrogen belowground and log_10_ total biomass for burned trees (r = 0.49, F = 8.629, p = 0.007) but there was no significant relationship for control (unburned) trees (r = 0.1, F = 0.409, p = 0.528).

## Discussion

The clearest result from this study was that eastern redcedars are shade-intolerant. Shade intolerance has been recorded for eastern redcedars by other authors [52, 109–112]. Indeed, there is a light optimum for photosynthesis higher than the maximum photosynthetically active radiation level that they measured (1750 μmol m^-2^ s^-1^) [110]. However, these measurements were all done in old-field grasslands; whether there are differences in photosynthetic ability between forest and old-field eastern redcedars is not known, although differences in phenotypes have been recorded [62]. Eastern redcedars can survive beneath a mature forest ([109]; pers. obs.). Interestingly, there was also a significant negative effect of shade on RGR diameter growth (but not height) of post oaks *Q*. *stellata*, a species usually considered to occur later in succession, when there is diminished light in forests [29, 113].

Light attenuation in forests can result in <10% sunlight reaching understory trees [111], yet eastern redcedars still survive [52]. It is likely that the longer growing season for these evergreen trees allows them to outcompete deciduous trees in the winter despite the lower light intensities [52]. I note that, although eastern redcedars are evergreen, they stop growing (even in the greenhouse in Ohio) over mid-winter when the mean low temperatures in January and February in neighboring Chardon are -9.8°C and -9.4°C, respectively.

There was no significant effect of soil quality (either fertilization or lime) on relative growth rate of eastern redcedars in the first experiment. This, at first glance, seems perplexing because it is known that eastern redcedars grow better in high-nutrient soils [62] and they are known to be common on limestone substrates [59–66]. Although the eastern redcedar is reputed to be able to tolerate a wide range of soil types, it has been widely observed that there are noticeable differences in their phenotypes on different soils [25, 26, 62]. High abundance of nutrients such as nitrogen may also be important for niche expansion of eastern redcedar, especially in old fields that were usually fertilized [20, 40, 42, 47, 49, 68, 69, 114]. Furthermore, a combination of shade and nitrogen fertilization may facilitate tree invasions of grasslands [115]. Possible reasons for the absence of an effect of nitrogen fertilization is that either there was sufficient nitrogen supplied by the potting soil, and/or that additional nitrogen would not further benefit growth (i.e., following Liebig’s law, there was another nutrient that was limiting [100]). However, several studies indicate that there is co-limitation of nutrients and not just nitrogen [116–118]. The purported preference for limestone substrates may reflect the fact that growing on cedar barrens may be related to the relative absence of interspecific competition there [64–66]. However, I found no effect of competition with post oaks, a common co-dominant [70], with the exception of a shade X lime X competition effect on belowground biomass of eastern redcedars. Experiments are currently underway to test whether eastern redcedars and post oaks, as well as grasses, are partitioning access to the water resource (Hamati et al., in prep.).

### Grass competition and fire

I found no support for the role of grass competition in the second experiment. A well-supported hypothesis to explain the coexistence of trees and grasses in the same habitat is the two-layer hypothesis [119]. Briefly, this hypothesis posits that grasses monopolize the upper soil layers while trees mostly use deeper water sources, even reaching as deep as the aquifer. A global meta-analysis has largely substantiated this hypothesis [120]. Many earlier studies have shown that there are significant negative effects for range-expanding trees of growing with grasses, which usually outcompete the woody plants [30, 119–128]. When rain falls, it is mostly taken up by shallow-rooted grasses. Some water percolates through to reach the deeper roots of trees. When grasses are heavily grazed, most frequently by domestic livestock, this frees up water for the trees to exploit. Heavy grazing might thus facilitate niche expansion by trees such as eastern redcedars because space, nutrients and moisture are provided to woody plants, leading to a rapid increase in abundance [36, 38, 102, 119, 124, 127, 128]. We [124] have also found that trees may grow their roots very quickly through the root layer occupied by grasses, minimizing the time that there is competition between grasses and trees. Another experiment clearly showed that absence of grazing resulted in low survival of eastern redcedar seedlings in grasslands, indicating that competition with herbaceous plants was important [36]. When grasses are removed by grazing animals, such as cattle, space is made available (and nutrients probably too) [7, 45, 119, 120, 125], and can often be occupied by eastern redcedar. I did not examine the effects of competition for water in either of these experiments, which was provided *ad libitum*. Fire may enhance the ability of the related redberry junipers *Juniperus pinchotii* in the Edwards Plateau of south-central Texas to outcompete grasses if fire and droughts occurred contemporaneously [38].

A possible reason for the absence of competition by grasses in the second experiment may be that the grasses were not sufficiently established to exert a competitive effect (they were planted one month prior to burning), especially by means of shading which is a common mechanism used by grasses to outcompete trees [42, 120, 129]. The most important negative effect of grasses was through a reduction in photosynthetically active radiation (PAR) and only secondarily by plant-available soil water, as indexed by soil clay content [42]. PAR has also been found to be of primary importance in grass-induced competition [130]. Because the grasses used in this experiment were relatively short, they were unlikely to have had a negative effect on eastern redcedars. However, I note that evidence for competitive effects of grasses on trees is mixed. Although there was a significant negative of grass competition [124, 126–128], precipitation has been found to be far more important than grass competition [8]. Both nutrients and water availability have been found to affect competition by grasses on tree seedlings [131]. For example, Bermuda grass (*Cynodon dactylon*) suppressed the growth of the sweet acacia tree (*Acacia farnesiana*; syn. *A*. *smallii*) in nutrient-rich soils but not in nutrient-poor soils [132]. The soils used in the second experiment were nutrient-rich, but no competitive effect of grasses or trees on eastern redcedars was observed. However, there may be little effect of herbaceous plant community diversity or composition on encroachment by eastern redcedars [51]. Competition for resources (i.e., nutrients) and non-resources (i.e., interference for space) could alter the effects of grasses on trees [133]. These authors found that early-successional forest species, such as eastern redcedars, were affected by both resource competition and non-resource competition in the form of non-self competition, while late-successional species, such as the post oak studied in the first experiment, were affected by resource competition only. I found very little effect of life-form competition (i.e., tree competition by post oaks on eastern redcedars or *vice versa*; with the exception of the aforementioned interaction between shade X lime X competition on belowground biomass of eastern redcedars) in the first experiment, even in fertilized soils.

Some researchers have found that niche- and range-expansion by eastern redcedar can be reduced by use of fire [37, 56, 134], although it may be necessary to treat individual trees [135]. In the second experiment reported on here, burned eastern redcedars were smaller after one month, were in poorer condition (Fig 4), and had lower nitrogen (but not total nonstructural carbohydrates) than unburned control trees. At least part of the reason for the negative effects of fire may be related to bark thickness. Bark thickness is known to be related to susceptibility to fire [122, 136–139]. Trees with thin bark, such as eastern redcedars, are more susceptible to fire [62]. In general, tree bark gets thicker as trees grow larger [136–138]. Thus, small trees such as those examined (Fig 4), are likely to be very susceptible to fire. The impact of the fire regime I applied was high [101, 102]. With less intense fires, it is less likely that larger, older, non-resprouting trees (such as the eastern redcedar [62]) would suffer mortality [10, 53, 126, 138]. Indeed, topkill (complete death of the aerial biomass) may be necessary to kill juvenile trees [140, 141], resulting in the ‘fire trap’ [142–144] that prevents them from recruiting into the adult size classes. However, in the experiment reported on here, there was a considerable number of young trees that did not die (Fig 4).

### Optimal partitioning theory

Unshaded plants had significantly higher concentrations of total non-structural carbohydrates (TNC) and lower concentrations of nitrogen than shaded plants (Fig 3). Shade tolerance is related to non-structural carbohydrate storage in plants [92]. To persist in the shaded understory, seedlings must maintain positive net carbon balance [92, 145, 146]. Furthermore, light availability in the understory is frequently close to the whole plant’s light compensation point, resulting in large potential consequences for seedling carbon balance [146]. Thus, the results indicating storage of non-structural carbohydrates reported on from the current study are consistent with those reported elsewhere. Another study found no relationship between aboveground productivity and carbon storage in 18 native and 21 non-native species from light-limited deciduous forest understories of Eastern North America in a common garden [147]. Contrastingly, in the current study, there was a significant difference in TNC due to shading the eastern redcedars, which were smaller in the shade treatment, although the other factors did not show any significant effects. Furthermore, larger eastern redcedars stored more TNC than small trees regardless of burning treatment. Thus, results are not generalizable to all understory species living in the shade [147, 148].

There was a significant effect of shade on the concentration of nitrogen (Fig 3), with a decrease in nitrogen concentration in unshaded plants. I did not find a significant effect of nitrogen fertilization on eastern redcedars in the first experiment. Nitrogen fertilization was found to have resulted in *higher* nitrogen content of mature needles of a conifer *Pinus radiata* [149]. Nitrogen fertilization also increased the proportion of nitrogen in the needles that was translocated to the new flush in this species [149].

Inconsistent effects of lime addition have been recorded in a meta-analysis of lime studies, perhaps due to differences in inherent preference of the target species for high pH soils [99]. I found a significant positive effect of lime on nitrogen concentration in the first experiment, in addition to a significant three-way interaction effect on belowground biomass between shade X lime X competition. My inference from the last-mentioned result is that, if the eastern redcedars are enduring competition from post oaks in shade, then lime is likely to have a negative effect. A similar effect of lime application on nitrogen concentration has been recorded [97]. They ascribed the increase in nitrogen concentration to an increase in the rate of mineralization of N by lime fertilization, due to increased microbial activity in lime-rich soils [98].

There were also significant negative effects of fire on growth of eastern redcedars in this study (Fig 4). I found a reduction in total nitrogen in burned eastern redcedars compared to unburned trees, which was due to a reduction in nitrogen belowground in burned trees (there was no difference between aboveground treatments). However, I found no difference in non-structural carbohydrates between burned and control trees. Contrastingly, a threefold decrease in total nonstructural carbohydrates (sugars and starches) was found in a resprouting range-expanding woody shrub *Cornus drummondii* after browsing and a prescribed fire in Kansas [3]. In another study, root starch was found to be a key nutrient that limits resprouting ability after fires in two savanna shrubs, *Miconia albicans* and *Clidemia sericea* [150]. However, root starch reserves were replenished in both species within two years after a burn [150], suggesting that only annual burns would cause a net loss of individuals. In the experiments reported on in the current study, there was a significant correlation between TNC and tree size, with larger trees storing more non-structural carbohydrates than smaller trees, regardless of treatment. This pattern also occurred for nitrogen in burned but not control trees. There was no significant differentiation in soluble sugars or starches with regard to the burning treatment. Thus, only the prediction regarding the negative effect of shading on TNC and elevated N with lime application were substantiated. Thus, optimal partitioning theory can only be considered to be partly supported.

## Conclusions and future directions

I conclude that shade has a far greater effect than fire in controlling the growth of eastern redcedars. Furthermore, the effects of fertilizer and lime additions were small, although the sustained long-term benefits for an eastern redcedar tree to be in a high-nutrient site or high lime sites are probably considerable. Interestingly, there were few effects of interspecific competition with a common co-dominant, the post oak, indicating that neither a preference for lime substrates nor avoidance of competition could explain their use of cedar barrens [57, 60, 66]. However, it is possible that avoidance of grass competition rather than competition with other trees may have been of importance. This result was not substantiated in the second experiment; there was no evidence of grass competition [130, 151], although perhaps the grasses could shade out the young seedlings, preventing their establishment, much in the same way as shade had a significant negative effect on the growth of young redcedars.

There may also be an interaction between nutrient availability and fire [42]; some of the oldest eastern redcedar trees (in excess of 500 years old) can be quite short (1.7–2.0 m) where they occur on low-nutrient soils in rocky areas where fires are rare, such as on the infertile soils of the Niotaze-Darnell complex on the steep cliffs in the Cross Timbers of Oklahoma, Arkansas, and Missouri [152]. In the Great Plains states of the U.S., the abundance of eastern redcedar in grasslands may be related to both heavy grazing and fire suppression [9, 10, 29, 152–156]. An important factor that may interact with the effects of fire and grazing may be the *timing* of fire, that was not tested in this experiment. When fires occur at the beginning of the wet season (i.e., before tree seeds are produced), then fire creates space in the grass sward for the tree seeds to germinate *en masse*, resulting in range expansion of woody plants [7]. However, if fires occur at the end of the wet season, those young tree seedlings that have managed to recruit during the wet season will be killed by the fire, and little or no range expansion will occur. These conclusions are consistent with those of Twidwell et al. (2016) [38] who found that fire interacted with drought in reducing the abundance of the eastern redcedar on the Great Plains. Future studies could manipulate root non-structural carbohydrates [157] to test the effects of storage on responses to grazing and fire.
